# HPV infection and triple-negative breast cancers: an Italian case-control study

**DOI:** 10.1186/s12985-014-0190-3

**Published:** 2014-11-21

**Authors:** Andrea Fausto Piana, Giovanni Sotgiu, Maria Rosaria Muroni, Paolo Cossu-Rocca, Paolo Castiglia, Maria Rosaria De Miglio

**Affiliations:** Hygiene and Preventive Medicine, Department of Biomedical Sciences, University of Sassari, Sassari, Italy; Department of Clinical and Experimental Medicine, University of Sassari, Sassari, Italy; Epidemiology and Medical Statistics Unit, Department of Biomedical Sciences, University of Sassari - Research, Medical Education and Professional Development Unit, AOU Sassari, Sassari, Italy; Surgical Pathology Unit, Department of Diagnostic Services, ASL Olbia, Olbia, Italy

## Abstract

**Background:**

Breast cancer is one of the most important neoplasia among women. To reduce its incidence and mortality impact it would be desirable to early identify risk factors associated with its development. It was recently suggested that biological agents could be the etiological cause, particularly Human Papilloma Virus (HPV). No specific relationship with different breast cancer types has been demonstrated until now. In particular, the triple-negative breast cancer (TNBC), characterized by a receptor negative pattern (ER/PgR/HER2–negative) and poor prognosis, can represent one of the most relevant clinical and public health priority in terms of observational research.

**Findings:**

Aim of the study was to evaluate the HPV-positivity prevalence in two breast cancer series (TNBC vs. non-TNBC) in Northern Sardinia, Italy. The sample size of each group was represented by 40 formalin-fixed and paraffin-embedded specimens. The mean age was 60.3 years. The majority of the cancers were ductal (84%). The grading distribution was different: G2 was the most prevalent grade in the non-TNBC series, whereas G3 was the most frequent in the TNBC series (70% and 72%, respectively). Six biological samples were HPV-positive (7.5%): the positivity was assessed only in the TNBC group (15%; p-value: 0.026). The isolated genotypes were: 16, 31, 45, 52, 6, and 66. Only one co-infection was found (i.e., HPV-6 and -66).

**Conclusions:**

The prevalence of HPV-positivity in TNBC specimens was 15%. On the basis of its carcinogenetic ability, an etiological role in the pathogenesis of the cancer could be supposed. This association should be confirmed with longitudinal studies to better assess the role of the HPV infection in TNBC and non-TNBC tumors.

## Findings

Breast cancer is the most common cancer and the principle cause of death from cancer in women worldwide [1]. Although reduction of global mortality might be achieved with early diagnosis selecting individuals on the basis of well-known risk factors (*i.e.*, age, familial history, and a previous history of breast cancer), the majority of the cases in low- and middle-income countries cannot be diagnosed with a secondary prevention strategy. Moreover, risk factors may be not recognized in a large proportion of patients [2]. Therefore, it is crucial to confirm the role of other suspected risk factors, such as viral infections due to Epstein-Barr virus, mouse mammary tumor virus, and human papilloma virus (HPV). To date, the relationship between HPV and breast cancer has not been fully elucidated: sexual transmission, by direct contact with genital region, and/or hematological spread [3] from genital lesions should be considered. The potential causal association between HPV infection and breast cancer was firstly described in 1992 [4]; however, some Authors confirmed the relationship in association with different HPV prevalence estimates [5-10] but others did not [11-13]. Incongruous findings may depend on the assay methods, HPV prevalence, and geographic differences [14-16]; besides, very few studies evaluated the association between HPV infection and breast cancer clinical-pathologic features, such as histologic subtypes, tumor grade, hormone receptors expression and Human Epidermal Growth Factor Receptor 2 (HER2) status [17-19].

In clinical practice, according to a recent molecular classification, immunohistochemical expression of Estrogen (ER) and Progesterone (PgR) receptors and HER2 allows to categorize breast cancers in different prognostic subgroups (luminal A, luminal B, HER2 overexpressing, and triple negative breast cancers [TNBC]), with distinct therapeutic approaches [20]. In particular, the TNBC phenotype (ER/PgR/HER2–negative), which accounts for 10-24% of invasive breast cancers, is characterized by earlier onset, poor prognosis, and limited therapeutic options [3], therefore, it may represent a priority epidemiological research target [21].

Recently, our group performed a case–control investigation, which described a high overall prevalence of HPV cervical infection in women living in Northern Sardinia, Italy. In particular, HPV types −16 and −51 DNA were detected in 12.5 and 6.3% of atypical squamous cells of undetermined significance; 24.0% and 16.0% of low-grade squamous intraepithelial lesions; 50.0% and 50.0% of high-grade squamous intraepithelial lesions, respectively [22]. Moreover, these figures were confirmed by a further retrospective study in embedded invasive cervical cancers, which highlighted the primary role of HPV-16 in the pathogenesis of cervical carcinoma followed by an unusual high prevalence of HPV-51 infection (36.2%) [23]. The recent availability of two HPV vaccines, recommended for the primary cervical cancer prevention, could imply the potential advantage of immunization practices in preventing other HPV-related cancers.

On this basis, we carried out a case–control study aimed at identifying the presence of HPV-DNA in embedded tissues series of TNBC diagnosed in women from Northern Sardinia.

Ethical approval and informed consent for this study was unnecessary, according to the Italian legislation concerning the guidelines for the performance of observational studies (G.U. n. 76. 31-3-2008). However, a formal approval of the study protocol was requested in 2013 to the Ethical Committee of the Azienda Sanitaria Locale n°1 of Sassari, Italy (PN-1137/L 2013).

A case–control study, based on a case–control design, was performed to evaluate the prevalence of HPV infection in a subset of 40 embedded TNBC and 40 embedded non-TNBC, as controls; both groups of breast cancer series belonged to women admitted to a tertiary University Hospital located in Sassari, Italy.

An ad-hoc case report form was created to record demographic, histopathological and virological information. Data were anonymized in compliance with the Law Decree No. 196/2003, article 24 (Code for the protection of personal data).

Based on a sample size calculation, 40 formalin-fixed and paraffin-embedded (FFPE) tissues from TNBC specimens were consecutively and retrospectively selected from the pool of those collected in the Pathology Unit, Department of Clinical and Experimental Medicine, University of Sassari. The selection of every TNBC specimen was associated with a FFPE non-TNBC specimen, as control, paired by age (±5 years). The enrolled series was diagnosed by a pathologist who was blinded to the final diagnosis. The same methodological blinding procedure was followed for the virological analysis, carried out in triplicate at the WHO quality-assured laboratory of the Hygiene and Preventive Medicine Unit, Department of Biomedical Sciences, University of Sassari (http://www.who.int/biologicals/areas/vaccines/hpv_labnet/en/).

Ten consecutive sections, 10 micron in thickness, were cut under sterile conditions from representative neoplastic tissue blocks of 80 breast cancers, in order to obtain genomic DNA. Nucleic acids were extracted with a commercially available extraction kit (QIAamp DNA FFPE Tissue Kit, Qiagen, Hilden, Germany) in accordance with the manufacturer’s instructions. To obtain genomic DNA 10ul of RNase A (20 mg/ml, Rnase PureLink, Life Technologies) were applied directly to the silica membrane to digest contaminating RNA. The quantity and the quality of nucleic acids were assessed spectrophotometrically (260 nm, 260/280 and 260/230 ratios, spectrum 220–320 nm) using Nanodrop ND1000 (EuroClone, Milan, Italy). Positive and negative controls were represented by HPV-positive samples and water blanks, respectively HPV DNA sequences were detected with the INNO-LiPA HPV Genotyping Extra kit (Innogenetics, Gent, Belgium), based on the reverse hybridization principle, according to manufacturer’s instructions. The INNO-LiPA assay covers all currently known high-risk HPV genotypes, such as high-risk (HPV 16, 18, 26, 31, 33, 35, 39, 45, 51, 52, 53, 56, 58, 59, 66, 68, 73, 82), low-risk HPV genotypes (HPV 6, 11, 40, 43, 44, 54, 70), and some additional types (HPV 69, 71, 74), while HPV 13, 34 and 67 genotypes are interpreted as HPV X. The INNO-LiPA assay utilizes a cocktail of biotinylated consensus primers (SPF10) to amplify a 65-bp region within the L1 Open Reading Frame of multiple HPV types. The biotinylated PCR products are then genotyped by hybridization to HPV type-specific oligonucleotide probes bound to nitrocellulose membranes and detected by an alkaline phosphatase-streptavidin conjugate and colorimetric detection. Post-PCR hybridization and colorimetric detection were performed using the Auto-LiPA 48 instrument in accordance with the manufacturer’s recommendations. At the end of color development, the strips were scanned and evaluated by the Line Reader and Analysis software (LiRAS) to determine if defined bands for a particular genotype probe were visible. A biological sample is considered HPV positive if at least one of the type-specific lines or one of the HPV control lines is positive. An additional primer pair for the amplification of the human HLA-DPB1 gene is added to monitor sample quality and extraction. When positivity for HR genotypes −16, −18, −31, −45, −51, and −52 was found, confirmation was obtained testing biological samples with an “in-house” Real-Time quantitative TaqMan PCR assay [11,22].

The sample size, calculated considering a first type error of 0.05, based on a case control ratio of 1:1, and an estimated exposure prevalence of 0.134, computed pooling proportions of 7 studies conducted in Europe [12], with a statistical power of 0.8 for an OR≥4, required 40 cases. Qualitative variables were summarized with percentages and were statistically compared using the z-proportion hypothesis test. Chi-square or Fisher exact test was used when appropriate. A p-value <0.05 was considered statistically significant. Statistical analysis was carried out using the statistical software Stata 11.0 (StataCorp LP, College Station, Texas).

A total of 80 embedded breast cancer specimens were analyzed: 40 were TNBC and 40 were embedded non-TNBC, namely 38 “luminal A” and 2 “HER2 overexpressing” variants. The mean (standard deviation) age of the patients was 60.3 (14.2) years; in particular, it was 60.3 (15.8) and 60.3 (12.6) for TNBC cases and controls, respectively (p-value: 0.99). Patients’ characteristics and pathology features are described in Table 1.

**Table 1:** **Demographic, pathological, and virological characteristics of the selected cohort**

**Variables**		**n = 80**	**Triple-negative**	**Other receptor pattern**	**p-value**
			**(n = 40)**	**(n = 40)**	
Mean age (SD), years		60.3 (14.2)	60.3 (15.8)	60.3 (12.6)	0.99
Histotype, n (%)	Invasive ductal	67 (83.8)	31 (77.5)	36 (90.0)	0.11
	Invasive lobular	5 (6.3)	2 (5.0)	3 (7.5)	
	Others	8 (10.0)	7 (17.5)	1 (2.5)	
Grading, n (%)	1	6 (7.6)	0 (0.0)	6 (15.0)	<0.0001
	2	39 (49.4)	11 (28.2)	28 (70.0)	
	3	34 (43.0)	28 (71.8)	6 (15.0)	
HPV+, n (%) [CI 95%]		6 (7.5) [1.7-13.3]	6 (7.5) [1.7-13.0]	0 (0.0) [0.0-0.0]	0.026

SD: Standard Deviation.

CI: Confidence Interval.

According to histopathological classification, the majority of the cancers were ductal (67, 83.8%), followed by lobular (5, 6.3%), and others (8, 10%). The proportional grading distribution in the entire series was the following: G_1_ 7.6% (n = 6), G_2_ 49.4% (n = 39), and G_3_ 43.0% (n = 34); in particular, G_1_ 0.0% (n = 0), G_2_ 28.2% (n = 11), and G_3_ 71.8% (n = 28) for the TNBC cases; conversely, the proportion for the non-TNBC controls was G_1_ 15.0% (n = 6), G_2_ 70.0% (n = 28), and G_3_ 15.0% (n = 6) (p-value <0.0001).

Out of 80 patients six (7.5%, 95% confidence intervals: 1.7-13.0) were positive for at least one HPV genotype. In particular, HPV positivity was detected only among the 40 TNBC (15.0%; p-value = 0.026). The most frequent genotypes were HPV-16 (28.6%), −31 (14.3%), −45 (14.3%), −52 (14.3%), −6 (14.3%), −66 (14.3%); the only co-infection detected was HPV-6 and HPV-66.

Logistic regression analysis did not identify covariates associated with HPV-positivity (results not showed).

HPV genome was detected in approximately 8% of the cases suggesting a viral pathogenesis of the cancer on the basis of its well known role in the carcinogenesis of the epithelial cells [14], even if the proportion found was below the average value assessed by other Authors [16,19]. However, the study we performed was not designed to evaluate the prevalence, being a case–control study, but to focus on the relationship between HPV infection and TNBC and to demonstrate its potential etiopathogenetic role in this kind of tumors.

The difference in terms of cellular differentiation was striking: the proportion of G3 was significantly higher in the TNBC group than in the non-TNBC category. However, no differences for the histological type were found, since the majority of the carcinomas showed ductal differentiation.

TNBC were the most undifferentiated carcinomas: they were the only neoplastic type found positive to the molecular analysis aimed at diagnosing HPV DNA. The non-TNBC series were HPV negative, and the difference in terms of positivity was statistically significant.

A more prevalent genotype was not identified: those detected were genotypes usually identified in the geographical context where this epidemiological study was carried out [22,23].

Even if the study carried out in our center detected only HPV-positivity in the TNBC, the probability of virological positive results in other cancers showing different receptor patterns could be hypothesized (e.g., [10]). Nevertheless, other studies confirmed that HPV-positivity can be frequently found in estrogen-receptor negative tumors or in those characterized by a high proliferation rate [9].

At our best knowledge, our research is one of the first observational studies proving an association between TNBC phenotype and HPV-positivity. Moreover, our findings strengthen the hypothesis of an association between HPV-positivity and poorly differentiated variants. Therefore, our results, which can represent the basis for future studies, should be carefully evaluated because only a few HPV positive cases were identified and other studies found HPV in breast tissues with estrogen-receptor expression [24]. Moreover, the low viral load, detected in several studies [25,26], can explain the low frequency of HPV-positive series.

Furthermore, the explanation of the role played by the host susceptibility will be necessary, proving if the genetic background can predispose to an imbalance of the native and/or adaptive immunity favoring the infection in that specific anatomical area, as well as the activation of an uncontrolled proliferation cycle.

The demonstration of the etiologic role of the HPV infection could be relevant, particularly because of the availability of highly efficacious preventive vaccines; in case of a research concordance on the role of HPV, this primary prevention strategy could increase the armamentarium of the current tools implemented in the breast cancer prevention.

This study shows some limitations. Its retrospective nature cannot assess if HPV infection precedes the cancer development. However, the epidemiological nature of the study, *i.e.* the case/control design, is the one suggested in case of rare events. The sample size is smaller than the statistical population size but, in order to avoid unreliable results related to unpowered studies, we computed the hypothesized sample size representative of the current population on the basis of several epidemiology-based assumptions. Furthermore, we could not assess the pathogenetic role of the HPV genotypes: new molecular hybridization in situ studies are needed to evaluate the cellular location and the pattern of hybridization of the HPV genome, that is to evaluate if the HPV-DNA is integrated in the human DNA or shows episomal replication in the neoplastic nuclei. It is straightforward that the integration in the human genome can alter the control of the cellular duplication cycle. Another study we are performing will try to answer to that relevant pathogenetic issue. The risk of a cross-contamination was avoided adopting sterile procedures during the preparation of the biological samples; the methodological efficacy of the molecular procedures was indirectly demonstrated by the detection of six different genotypes out of six positive TNBC samples.

Another unsolved problem is the origin of the HPV: to understand the original site of infection is important for the identification of preventive measures. On this basis, a concomitant virological study on cervical and/or oral-laryngeal samples could have allowed to appreciate if the sexual transmission has a role in the human spread of HPV types [14]. Unfortunately, the missing systematic cervical cancer screening of the patients in Sardinia did not allow the detection of HPV infection in other anatomical sites for the individuals enrolled in our study.

It is clear that the immunization programs, carried out in young adults, could play a role in the near future in the protection against those genotypes involved in the cancer pathogenesis and included in the currently available vaccines. However, the low prevalence of HPV suggests that other epidemiological, demographic (e.g., genetic background), clinical, virological risk factors should be assessed to better understand the potential synergistic or additive pathogenetic role of HPV genotypes.

## Abbreviations

HPV: human papilloma virus
HER2: Human Epidermal Growth Factor Receptor 2
ER: Estrogen receptor
PgR: Progesterone receptor
TNBC: Triple-negative breast cancer
FFPE: formalin-fixed and paraffin-embedded
LiRAS: Line Reader and Analysis software
